# Axon Initial Segment Cytoskeleton: Architecture, Development, and Role in Neuron Polarity

**DOI:** 10.1155/2016/6808293

**Published:** 2016-07-17

**Authors:** Steven L. Jones, Tatyana M. Svitkina

**Affiliations:** Department of Biology, University of Pennsylvania, Philadelphia, PA 19104, USA

## Abstract

The axon initial segment (AIS) is a specialized structure in neurons that resides in between axonal and somatodendritic domains. The localization of the AIS in neurons is ideal for its two major functions: it serves as the site of action potential firing and helps to maintain neuron polarity. It has become increasingly clear that the AIS cytoskeleton is fundamental to AIS functions. In this review, we discuss current understanding of the AIS cytoskeleton with particular interest in its unique architecture and role in maintenance of neuron polarity. The AIS cytoskeleton is divided into two parts, submembrane and cytoplasmic, based on localization, function, and molecular composition. Recent studies using electron and subdiffraction fluorescence microscopy indicate that submembrane cytoskeletal components (ankyrin G, *β*IV-spectrin, and actin filaments) form a sophisticated network in the AIS that is conceptually similar to the polygonal/triangular network of erythrocytes, with some important differences. Components of the AIS cytoplasmic cytoskeleton (microtubules, actin filaments, and neurofilaments) reside deeper within the AIS shaft and display structural features distinct from other neuronal domains. We discuss how the AIS submembrane and cytoplasmic cytoskeletons contribute to different aspects of AIS polarity function and highlight recent advances in understanding their AIS cytoskeletal assembly and stability.

## 1. Introduction

Neurons are highly polarized cells that form the basis of directed information flow within the nervous system. Morphologically, each neuron consists of two distinct domains. On one end, the somatodendritic domain contains the cell body (soma), multiple dendrites, and a short region of the proximal axon adjacent to the soma (axon hillock), while the axonal domain projects a long axon away from the axon hillock toward the next neuron in the neural circuitry or toward a target tissue where it branches into multiple axon terminals that form synapses. Functionally, information flow commences at the somatodendritic domain, which receives synaptic input from neighboring or distant neurons, and is subsequently transmitted to the axonal domain, which specializes in sending signals to the next neuron in the neural circuitry or to a target tissue.

The functional and morphological polarity of neurons depend on the asymmetric distribution of specific molecules to the axonal and somatodendritic domains. The establishment of molecular asymmetry between these domains is fundamental to axon specification during early neuron development. However, for the long-term maintenance of neuronal function and shape, axons and dendrites must retain their unique molecular components throughout the life of the organism. The axon initial segment (AIS) is a specialized compartment in neurons that separates the axonal and somatodendritic components. The AIS is uniquely positioned in the proximal axon, adjacent to and distal from the axon hillock, where it serves two functions: (1) to integrate synaptic inputs and generate action potentials and (2) to maintain neuron polarity. The roles of the AIS in action potential initiation and synaptic input integration have been discussed extensively in recent reviews [1–4] and will not be reviewed here.

The AIS contributes to the maintenance of neuron polarity in at least two ways: first, it serves as a submembrane diffusion barrier that restricts the mobility of plasma membrane components, preventing them from passing from one domain to another [5, 6]; and second, it acts as an intracellular selective filter for the transport of organelles and molecules between these domains through the cytoplasm. Together, the diffusion barrier and intracellular selective filter help to successfully maintain neuron polarity, and this success is partly due to different aspects of the AIS cytoskeleton.

In addition to the AIS, some neurons contain nodes of Ranvier, the small periodic gaps in the myelin sheath that surrounds the axon. Nodes of Ranvier are partly responsible for the astonishing speed and propagation of signal transmission along the axon. Although nodes of Ranvier share many features with the AIS in terms of protein composition and ability to generate action potentials for saltatory nerve conduction, nodes have not been implicated so far in polarity-related functions and will be discussed only minimally to point out some key similarities or differences relative to AIS structure. For recent detailed reviews on the assembly and maintenance of nodes, see [7, 8].

The overall goal of this review is to discuss current understanding of AIS cytoskeletal organization and its role in AIS function. We start with an overview of AIS structure, followed by a more in-depth review of AIS composition and architecture, and how certain features of AIS cytoskeletal structure underpin its role in neuron polarity. We end with a discussion of mechanisms of AIS cytoskeletal assembly and maintenance.

## 2. Overview of AIS Structure

**Figure 1:**
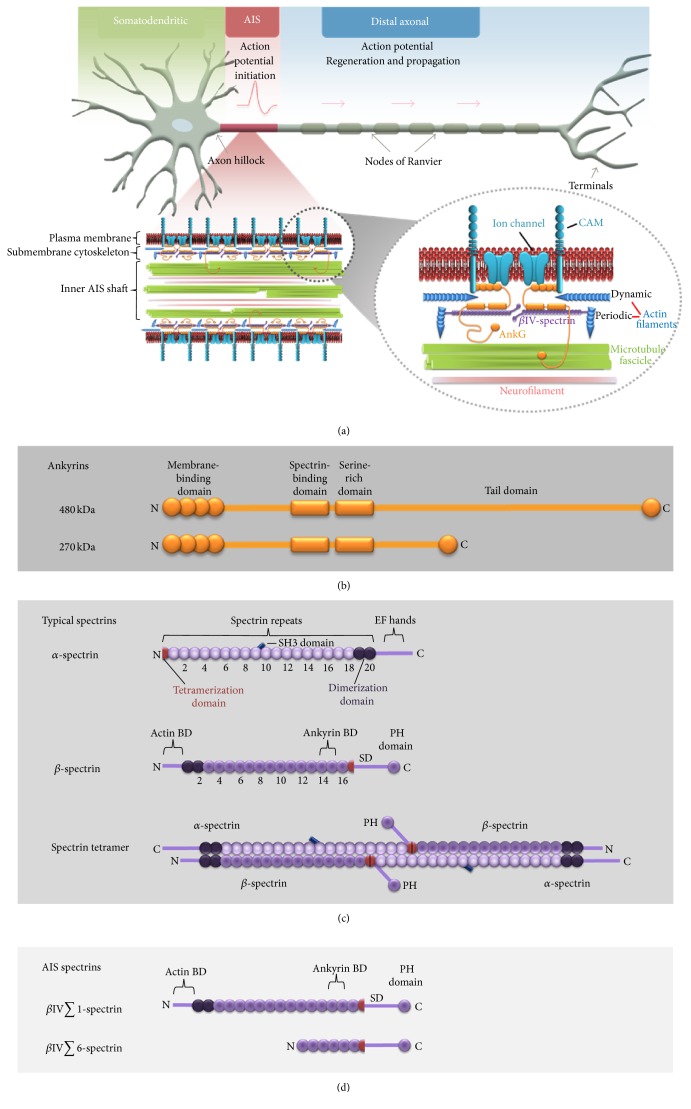
Architecture of the axon initial segment (AIS) and its key protein components. ((a), top) Neuron polarity. Polarized neurons receive synaptic inputs in the somatodendritic domain (green), which transmits the signals through the axon hillock to the axon initial segment (red). The AIS integrates synaptic inputs and initiates an action potential that gets propagated along the distal axon (blue) and amplified at nodes of Ranvier. ((a), bottom) Molecular organization of the AIS. The AIS can be divided into three layers: the plasma membrane, submembrane cytoskeleton, and inner AIS shaft (left), each having AIS-specific features (zoomed view at right). The scaffolding protein ankyrin G (AnkG) recruits many other proteins to the AIS and can interact with components in the different AIS regions. In the plasma membrane, AnkG through its N-terminal membrane-binding domain binds voltage-gated ion channels, which are important for action potential initiation and regulation, and cell adhesion molecules (CAMs). The submembrane cytoskeleton contains AnkG, *β*IV-spectrin, and actin filaments. These proteins form a periodic network along the entire length of the AIS. Periodic actin is spaced ~190 by at least two *β*IV-spectrin subunits, which in turn attach to the membrane through interactions with AnkG. In addition to periodic actin, relatively long, randomly oriented, dynamic actin filaments also exist in the submembrane cytoskeleton, and these filaments may have functions distinct from periodic actin. The inner AIS shaft contains microtubules bundles (fascicles), neurofilaments, and potentially also actin filaments (not shown). AnkG can extend its C-terminal tail into the inner AIS shaft where it is predicted to interact with microtubule fascicles. (b) Domain organization of isoforms of ankyrin G (AnkG). AnkG population contains two large neuron-specific isoforms, 270 kDa and 480 kDa, that localize specifically to AIS and nodes of Ranvier. The N-terminal membrane-binding domain of AnkG contains 24 ANK repeats (33-amino acid motif that mediates protein interactions). These 24 ANK repeats fold to form four independent subdomains (each containing 6 ANK repeats) that compose a globular membrane-binding domain. The spectrin-binding domain allows interactions with *β*IV-spectrin and thereby attachment of the submembrane cytoskeleton to the membrane. The serine-rich domain is glycosylated with N-acetylglucosamine monosaccharides. 480 kDa AnkG contains a 220 kDa insert following the spectrin-binding domain that is predicted to form a random coil. (c) Domain organization of typical *α*- and *β*-spectrin isoforms. (Top) *α*-spectrin comprises an incomplete spectrin repeat at the N-terminus (red), 20 complete spectrin repeats (violet), an SH3 (Src homology 3) domain (dark blue) inserted into spectrin repeat 9, and two EF-hand motifs at the C-terminus. Last two spectrin repeats (dark violet) can interact with the first two spectrin repeats in *β*-spectrin to form an antiparallel dimer. (Middle) *β*-spectrin comprises an N-terminal actin-binding domain (BD), 16 full spectrin repeats (violet), an incomplete 17th spectrin repeat (red), a variable region specific for individual *β*-spectrin isoforms (specific domain, SD), and a C-terminal pleckstrin homology (PH) domain. Spectrin repeats 1 and 2 (dark violet) dimerize with *α*-spectrin; spectrin repeats 14 and 15 interact with AnkG. (Bottom) A typical spectrin molecule represents an *αβ*-heterotetramer (two *α*- and two *β*-subunits) that form a rod-shaped structure. Two *αβ*-spectrin dimers, each formed by transverse interaction between *α*-spectrin repeats 19 and 20 and *β*-spectrin repeats 1 and 2, associate with each other longitudinally by making complete spectrin repeats through pairwise interaction of the incomplete spectrin repeats at the N-terminus of *α*-spectrin and the C-terminus of *β*-spectrin. (d) AIS-specific *β*IV-spectrin isoforms. *β*IV∑1 and *β*IV∑6 spectrins. The full-length *β*IV∑1 isoform has organization typical to *β*-spectrins (c). The *β*IV∑6 isoform lacks the N-terminus and first 10 spectrin repeats.

The AIS occupies about 20–60 *μ*m of the proximal axon length. The structure of the AIS was first investigated by early electron microscopy studies using thin sections of brain tissue [9, 10]. These studies demonstrated two distinct features of AIS structure: a dense plasma membrane undercoat and microtubule fascicles, which localize more deeply within the AIS cytoplasm. Thus, when viewed in cross section, the AIS can be divided into three main layers or regions: the plasma membrane (outermost surface), submembrane cytoskeleton corresponding to the plasma membrane undercoat (middle layer), and inner AIS shaft (cytoplasmic region) (Figure 1). These layers are integrated by a multidomain scaffolding protein ankyrin G (AnkG) that functions as a master organizer of the AIS.

The plasma membrane of the AIS is different from that of the axonal domain (herein also referred to as the distal axon) because it is enriched with specialized transmembrane proteins, including voltage-gated ion channels, which confer the unique electrical properties of the AIS and nodes of Ranvier, and specific cell adhesion molecules (CAMs).

The submembrane cytoskeleton plays a crucial role in AIS polarity function because disrupting its structure compromises axon identity. Its protein composition was suggested by light microscopy studies that revealed accumulations of several membrane-binding and cytoskeletal proteins in the AIS. The major cytoskeletal components that are currently known to compose the submembrane AIS cytoskeleton include actin filaments, the master organizer of the AIS ankyrin G (AnkG), and *β*IV-spectrin. The initial idea about organization and functions of this set of proteins came from studies of erythrocytes, where these or related proteins form a highly stable and elastic cytoskeletal network [11–15] that is important for conferring mechanical stability and resilience to cells as they travel through blood vessels [11]. It is now known that a similar ankyrin-spectrin-actin network, though with different geometry, composes the submembrane cytoskeleton of the AIS and distal axon [16–20] and may have functions comparable to those in erythrocytes [21].

The cytoplasmic region of the AIS, the inner AIS shaft, contains three major cytoskeletal filaments: neurofilaments, microtubules, and actin filaments. These cytoskeletal components are ubiquitous in neurons and contribute to various aspects of neuronal morphology, integrity, and function [22–24]. In the AIS, these filaments are thought to have additional roles in contributing to different aspects of AIS function.

## 3. Ankyrin G

Because of its reputation as the master organizer, AnkG may be considered the most important AIS constituent. In mammals, ankyrins are encoded by three different genes, ankyrin R (ANK1), ankyrin B (ANK2), and ankyrin G (ANK3), all of which have similar structure and function, and undergo alternative splicing to generate multiple isoforms [25]. Two large AnkG isoforms, 270 kDa and 480 kDa, are neuron-specific and localize particularly within the AIS and along the distal axon in nodes of Ranvier [26, 27]. The role of AnkG as a master organizer is attributable to the observations that it is the first protein that clusters in the proximal axon following axon specification, recruits almost all other AIS proteins to the plasma membrane and submembrane regions [28–31], and interacts with components within all layers of the AIS [11, 16].

The multifaceted activity of AnkG in the AIS depends in part on the various domains of the AnkG molecule. Starting at its N-terminus, AnkG contains a membrane-binding domain consisting of 24 ankyrin repeats (33-amino acid motifs involved in protein recognition), followed by a spectrin-binding domain, a serine-rich domain, a long, likely unstructured, tail, and a C-terminal domain [32] (Figure 1). AnkG associates with components in the plasma membrane and with spectrin in the submembrane region through its membrane-binding and spectrin-binding domains, respectively. The AnkG C-terminal tail extends to the inner AIS shaft [16], where it possibly associates with other interaction partners. For example, AnkG is thought to interact with microtubules and bundle them [11], which may lead to the formation of microtubule fascicles in the cytoplasmic region of the AIS, although direct interactions between AnkG and fascicles in the AIS have yet to be shown.

AnkG is indispensable for the localization and retention of other AIS proteins. Its depletion in neurons disrupts or prevents the formation of the AIS (and nodes of Ranvier). AnkG deficiency also contributes to the pathology of several neurological diseases (e.g., epilepsy, schizophrenia, bipolar disorder, autism spectrum disorder, and Alzheimer's disease) [33, 34]. Mutant mice lacking AnkG in their cerebellum develop severe ataxia and an inability to initiate action potentials, likely due to the observed loss of voltage-gated sodium channels at the AIS of Purkinje neurons [28, 29].* In vitro*, Purkinje and hippocampal neurons lacking AnkG exhibit a similar loss of sodium channels and other AIS proteins [29, 35, 36]. Thus, AnkG has a key role in AIS development and stability and is required for the long-term health of the nervous system.

## 4. AIS Plasma Membrane

The main proteins specifically accumulating in the AIS plasma membrane include ion channels and cell adhesion molecules (CAMs) of the L1 family. These transmembrane proteins are recruited to the AIS plasma membrane through interactions with the N-terminal membrane-binding domain of AnkG.

AIS-enriched ion channels include voltage-gated sodium (Nav), potassium (Kv), and calcium (Cav) channels. Accumulation of Nav channels in the AIS is essential for the local inward ion current that generates action potentials [37]. Nav channels consist of four transmembrane domains (numbered I–IV), each of which comprises six membrane-spanning segments. Nav channels bind AnkG through an AIS-targeting motif in the cytoplasmic loop between transmembrane domains I-II [38, 39]. The major Nav channel subtypes enriched in the AIS include Nav 1.6, Nav 1.2, and Nav 1.1, but the specific assortment of these channels and their longitudinal distribution along the AIS (e.g., distal versus proximal) depend on the specific type and developmental stage of the neuron [40–42]. The observation that some channel subtypes can be asymmetrically distributed along the AIS underscores the notion that the AIS itself can be further subcompartmentalized along its length. The mechanisms contributing to this longitudinal asymmetry apparently include additional targeting determinants besides binding to AnkG, because most AIS transmembrane proteins, including the Nav channel subtypes, bear a similar AnkG binding site. However, these mechanisms are currently unknown.

Potassium channel clustering at the AIS is essential for regulating action potential firing because it suppresses neuron excitability [31, 43]. Kv7.2 and Kv7.3 (KCNQ2/3) bear an AIS-targeting motif similar to that of Nav channels and accumulate as both homomeric Kv7.2 and heteromeric Kv7.2/7.3 complexes [31]. Kv1.1 and Kv1.2 channels are also enriched at the AIS. However, unlike Kv7.2 and Kv7.3, they do not contain an identified ankyrin-binding motif. Instead, Kv1.1 and Kv1.2 channels bind in the distal AIS to the synaptic scaffolding protein PSD-93 [44, 45], which is itself enriched there through an unknown mechanism. Kv1.1 and Kv1.2 function in conjunction with the calcium channels Cav2 and Cav3 (which are distributed throughout the AIS) to modulate action potential initiation [1].

In addition to ion channels, the L1 CAMs neurofascin 186 (NF-186) and neuronal cell adhesion molecule (NrCAM) accumulate at the AIS in an AnkG-dependent manner [11]. Both NF-186 and NrCAM are single-pass transmembrane proteins that contain a long chain of fibronectin and immunoglobulin repeats in their extracellular regions and bind AnkG via a highly conserved sequence (FIGQY) on their cytoplasmic domains [46]. Interestingly, a single AnkG molecule can accommodate at least two CAMs and one ion channel simultaneously [11]. The significance of CAMs at the AIS may involve linking the submembrane AIS cytoskeleton to structures outside the AIS, such as the extracellular matrix or other cells. For instance, NF-186 in hippocampal neurons recruits components of the AIS extracellular matrix (e.g., brevican) [36]. Furthermore, NF-186 regulates formation and maintenance of GABAergic synapses between the AIS of Purkinje neurons and axonal terminals of basket interneurons [47]. These axon-AIS synapses are important for modulating synaptic integration and action potential initiation.

Due to the protein-protein interactions between the plasma membrane and submembrane regions, the components of the submembrane cytoskeleton (e.g., AnkG and *β*IV-spectrin) regulate not only the recruitment and retention of ion channels and CAMs, but also their organization [48]. In addition, the ability of ion channels and CAMs to be anchored to the cytoskeletal network contributes to the role of AIS in neuron polarity, not only to the electrical activity of the plasma membrane (discussed further below).

## 5. AIS Submembrane Cytoskeleton

### 5.1. Protein Composition of the AIS Submembrane Cytoskeleton

The region of the AIS immediately beneath the plasma membrane contains the cytoskeletal proteins actin and *β*IV-spectrin, as well as the N-terminal segment of the AnkG molecule that interacts with transmembrane proteins and *β*IV-spectrin.

Spectrin molecules typically represent *αβ*-heterotetramers (two *α*- and two *β*-subunits) that form 150–200 nm long flexible rod-shaped structures. *α*-spectrin subunits typically consist of one incomplete spectrin repeat at the N-terminus, 20 complete spectrin repeats, an SH3 (Src homology 3) domain inserted into spectrin repeat 9 (also known as spectrin repeat 10 for historical reasons), and a C-terminal domain containing two EF-hand motifs, only one of which can bind calcium (Figure 1(c)). Classical *β*-spectrins comprise an N-terminal actin-binding domain, followed by 16 tandem full spectrin repeats, an incomplete 17th spectrin repeat, a variable region specific for individual *β*-spectrin isoforms (also known as the specific domain), and a C-terminal pleckstrin homology (PH) domain (Figure 1(c)). Within each spectrin tetramer, the incomplete spectrin repeats at the N-terminus of an *α*-subunit and the C-terminus of a *β*-subunit interact to form a complete spectrin repeat, forming a parallel longitudinal heterodimer that, in turn, associates with the second dimer in an antiparallel manner through lateral interaction between the first two *β*-spectrin repeats and the last two *α*-spectrin repeats (Figure 1(c)) [49]. Accordingly, the two *β*-spectrin subunits within tetramers orient with their N-terminal actin-binding domains at the two ends, where they can interact with actin filaments, and their C-termini facing each other, but not directly interacting, at the center of the molecule. *β*-spectrin interacts with AnkG through repeats 14-15 [35, 50–53]. Thus, both ankyrins and actin filaments can interact with spectrin molecules by binding to the *β*-spectrin, not the *α*-spectrin, subunit. In the membrane cytoskeleton of erythrocytes, the spectrin molecules are cross-linked by short actin filaments at so-called junctional complexes into a polygonal/triangular network, which gets tethered to the membrane primarily by ankyrin R, but also by components of the junctional complex [11–15].

In mammals, *β*-spectrins are encoded by five different genes, but only *β*IV-spectrin has been found to specifically localize to the AIS in mature neurons [11]. The proline-rich specific domain (SD) of *β*IV-spectrin between the 17th spectrin repeat and the PH domain has been hypothesized to confer specific functionality to *β*IV-spectrins, possibly to allow the recruitment of unknown proteins to the AIS or nodes of Ranvier [30]. The *β*IV-spectrin gene is alternatively spliced to generate six isoforms (*β*IV∑1–*β*IV∑6) [30, 54]. Two of these isoforms, *β*IV∑1 and *β*IV∑6, are known to specifically cluster at the AIS and nodes of Ranvier in both peripheral and central nervous system neurons, whereas other *β*IV-spectrin isoforms do not [54, 55]. Initially, *β*IV∑1-spectrin was suggested to be most important since it is the only AIS isoform containing an actin-binding domain, and its selective depletion from neurons causes AIS and nodal disappearance [55]. However, the truncated *β*IV∑6 isoform, which lacks the N-terminus and the first 10 spectrin repeats [54, 55] (Figure 1(d)), was shown to be more highly expressed relative to *β*IV∑1 [30] and specifically involved in Nav channel stability at the AIS and nodes of Ranvier [56]. This suggests that both isoforms may contribute to maintenance of AIS membrane and cytoskeletal proteins. However, the truncated *β*IV∑6 isoform does not rescue the membrane instability (detached wavy membranes) resulting from the absence of *β*IV∑1 [56] and is not expected to interact with actin filaments. Therefore, it is still unclear how *β*IV∑6 spectrin contributes to cytoskeletal function.

There are only two *α*-spectrin genes in mammals, *α*I-spectrin that is preferentially expressed in erythroid cells and ubiquitous *α*II-spectrin, both of which undergo alternative splicing to produce a variety of isoforms. *α*I- and *β*I-spectrin subunits are the major components constituting the membrane skeleton of erythrocytes and are also expressed in a subset of neurons [57]. Neurons also express *α*I/*β*I∑2 and *α*II/*β*II∑1 (known as brain spectrin) subunits that, respectively, localize to the somatodendritic domain and primarily to the distal axon [58, 59]. In Purkinje neurons, *β*III-spectrin is preferentially expressed in the somatodendritic domain [60]. Surprisingly, it remains unclear whether any *α*-spectrin is present in the AIS. Although *β*IV∑1 and *β*IV∑6 can be immunoprecipitated from brain lysate by an *α*II spectrin antibody [56], immunolocalization data supporting the presence of *α*II spectrin in the AIS are currently unavailable.

Actin filaments are thin (~7 nm diameter) helical polymers made of globular actin subunits. Actin filaments are dynamic and polarized, with polymerizing barbed ends and depolymerizing pointed ends. For some of their functions, actin filaments exist as stable polymers with fixed lengths due to association of actin-binding proteins along the length and at the ends of the filaments. For instance, in junctional complexes of erythrocytes, actin filament length and stability are controlled, in part, by the pointed- and barbed-end capping proteins tropomodulin and adducin, respectively, and by tropomyosin along the length [15].

Actin filaments were initially thought to be highly enriched in the AIS and form the bulk of the diffusion barrier responsible for polarity [61, 62]. However, later studies revealed that although actin filaments are present in the AIS, they are only minimally enriched there relative to the distal axon [19]. These filaments are typically assumed to be relatively stable, similar to stabilized actin filaments in erythrocytes. However, very little is known about the factors determining the stability of actin filaments in the AIS. To date, neither tropomodulin nor tropomyosin has been identified in the proximal or distal axon. The barbed-end capping protein adducin, on the other hand, is present and colocalizes with the axonal actin throughout neuron development. However, the cytoskeleton-associated population of adducin in the proximal axon of young neurons is progressively lost from the AIS as neurons mature [19]. Also, it is unclear to what extent *β*IV-spectrin, or any other protein that may associate along the filament length in the AIS, contributes to actin filament stability. Therefore, mechanisms of actin stabilization in the AIS remain an open question.

### 5.2. Architecture of the AIS Submembrane Cytoskeleton

**Figure 2:**
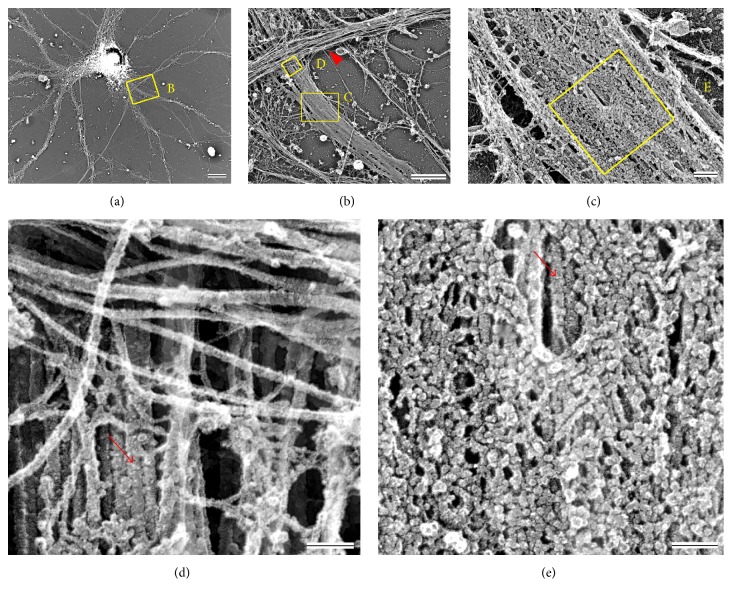
Platinum replica electron microcopy (PREM) revealing axon initial segment (AIS) structure of 21 DIV hippocampal neuron. (a) A neuron with dendrites and an axon (box B) extending from the soma. (b) Enlargement of box B in (a) showing a segment of the AIS (box C) and a portion of the axon hillock (box D), a region of the neuron that connects the soma and axon. The axon hillock is largely covered by an axon or dendrite (arrow head) from a different neuron. (c) Enlargement of box C in (b). (d) Enlargement of box D in (b) showing a microtubule fascicle (arrow) in the axon hillock that appears to enter the AIS. Most of the microtubules in the axon hillock are nonfasciculated. (e) Enlargement of box E in (c) showing a dense coat of globular and fibrillary structures. This coat corresponds to two upper layers of the AIS cytoskeleton, the immobilized plasma membrane proteins and submembrane cytoskeleton. Many of the globular structures in the coat correspond to voltage-gated ion channels and AnkG bound to its interaction partners. The fibrils represent cell adhesion molecules (CAMs), *β*IV-spectrin, and some AnkG molecules lacking interacting proteins. A microtubule fascicle can be seen in an opening of the coat where globules and fibrils are lacking (arrow). Note that such occasional openings in the AIS coat create small windows through which the inner AIS shaft can be seen (see Figure 1(a)). Bars: (a) 10 *μ*m; (b) 2 *μ*m: (c) 200 nm; (d) 100 nm; (e) 100 nm.

The fine structure of the AIS undercoat, which by thin section electron microscopy appeared as a fuzzy layer underneath the plasma membrane, was recently resolved in great detail by platinum replica electron microscopy (PREM) (Figure 2) [19]. This study demonstrated a remarkable density, as well as apparent elasticity, of the submembrane AIS cytoskeleton and identified its key components at a macromolecular resolution using immunogold labeling. The overall picture emerging from this analysis is that the AIS cytoskeleton represents a sophisticated and highly integrated network of submembrane and transmembrane proteins that overlays microtubules located in the AIS interior. These findings provide structural evidence to support earlier ideas that AIS-enriched proteins detectable by light microscopy compose the membrane undercoat.

A complementary insight into organization of AIS components on a global scale was obtained using subdiffraction fluorescence microscopy. Similar to other fluorescence techniques, subdiffraction microscopy evaluates only one or two labeled proteins at a time, which significantly decreases complexity of obtained images. At the same time, it can visualize additional details compared with diffraction-limited approaches. Using Stochastic Optical Reconstruction Microscopy (STORM), it was revealed that circumferential actin filament signals (also called actin rings) are periodically distributed throughout the entire length of the axon including the AIS, and each actin ring is spaced ~190 nm apart from its neighbors (Figure 1) [16, 18, 20].

This spacing is thought to be created by spectrin molecules, because the length of typical *α*-spectrin/*β*-spectrin tetramers is ~190 nm. Indeed, C-termini of *β*-spectrins (*β*II in the distal axon and *β*IV in the AIS) were found to localize in between adjacent actin rings, consistent with their position in the middle of the spectrin tetramer, while actin-binding N-termini colocalized with actin rings. Visualization of *β*IV-spectrin in the AIS using 3D STORM showed that both N- and C-terminal domains of *β*IV-spectrin are located at the same plane within the submembrane cytoskeleton [16]. Although the easiest interpretation of these findings is that *β*IV-spectrin partners with an *α*-spectrin to form conventional heterotetramers, a complication arises from the fact that no *α*-spectrin has been identified so far in the AIS. One possibility is that *α*-spectrin is present in the AIS but remains to be detected. Another possibility is that one of the *β*IV-spectrin isoforms (possibly *β*IV∑6) somehow substitutes *α*-spectrin, although a biochemical mechanism for such substitution is obscure. It is also unclear how *β*IV∑6-spectrin is recruited to and arranges in the AIS. Given that the C-terminally directed *β*IV-spectrin antibody detects both *β*IV∑1 and *β*IV∑6 isoforms and exhibits long-range periodicity that alternates with actin rings, C-termini of both isoforms likely colocalize at the center between actin rings. The position of *β*IV∑6 N-terminus has not been investigated. It can align with *β*IV∑1 underneath the plasma membrane or extend away from the periodic network toward the plasma membrane where it can help regulate Nav channel clustering [56]. Since *β*IV∑6 can localize to the AIS in a *β*IV∑1-independent manner [56], it is probably positioned between adjacent actin rings by interactions with AnkG. It would be interesting to see whether *β*IV∑6 periodicity forms or remains in the absence of *β*IV∑1 isoform.

AnkG and ankyrin B (AnkB) were also observed by STORM to exhibit periodic localization between adjacent actin rings in the AIS and distal axon, respectively [20]. The periodicity and spacing of ankyrins likely depend on *β*-spectrin, since ankyrin binds *β*-spectrin close to *β*-spectrin's C-terminus. However, the periodicity of ankyrins is less robust compared with that of actin and *β*-spectrins, likely because each spectrin tetramer contains at least two ankyrin-binding sites, each located slightly off-center of the spectrin molecule, which may or may not be fully occupied [52, 53]. Consistently, AnkG binding partners in the AIS, such as ion channels, also exhibit only a roughly periodic organization [20]. Analysis of AnkG organization in the AIS using domain-specific antibodies showed that while the N-terminal spectrin-binding and serine-rich domains of AnkG both exhibit a periodic pattern, the C-terminal regions of AnkG display poor regularity [16, 18, 20]. This arrangement further confirms that *β*IV-spectin imposes periodicity on the AnkG N-terminus containing the spectrin-binding site, whereas the unstructured C-terminal tail of AnkG gradually loses its periodicity [16, 18] and exends internally into the AIS cytoplasm (discussed later).

There are also important outstanding questions relating to the nature of the periodic actin rings. By analogy with erythrocytes, actin within rings was suggested to represent short, stable filaments since they colocalize with adducin in the axon [20]. However, the actual length of such filaments remains unknown and their stability is questionable. Thus, a significant portion of actin is depleted from actin rings in the AIS after treatment with the actin depolymerizing drug latrunculin A, while the remaining actin still exhibits periodic organization [16]. In contrast, latrunculin A treatment completely disrupts actin periodicity in distal axons [18, 20]. Since adducin has been characterized as a weak barbed-end capping protein (relative to other barbed-end cappers, such as gelsolin or capZ) [63] and other actin-stabilizing proteins, such as tropomodulin and tropomyosin, have not yet been reported to localize to axonal rings, the partial stability of actin filaments in the axon is not too surprising. As for the AIS, maturation-dependent loss of adducin from this region [19] makes the state of actin filaments deduced from the STORM data even less certain.

Direct visualization of actin filaments by PREM after their selective labeling by myosin subfragment 1 or immunogold revealed that, in the AIS submembrane cytoskeleton, actin exists as two populations of filaments: relatively long (~330 nm) filaments that can be classified as dynamic because they are sensitive to both the actin depolymerizing drug latrunculin B in live neurons and severing by gelsolin in cytoskeletal preparations and short stable filaments that exhibit resistance to these treatments [19]. The correlation between actin populations detected by PREM and STORM is not clear. Submicrometer long actin filaments have not been explicitly reported by studies applying subdiffraction fluorescence microscopy, although they are visible in the AIS by wide field fluorescence microscopy as bright diffraction-limited puncta [19]. Although short stable actin filaments in PREM images seem to be good candidates to correspond to actin rings observed by subdiffraction techniques, they did not display obvious periodicity in PREM images of the AIS. Zhong et al. reported that the detergent extraction procedure used for PREM preparation disrupted periodic actin organization [18]. Considering an ability of the PREM preparation procedure to preserve even dynamic actin filaments in the AIS [19], it is odd that it causes a significant loss of actin filaments from the rings that are assumed to be stable, although a partial loss cannot be excluded. It is also possible that the actin-spectrin network acquires periodic organization in response to longitudinal tension, which exists in living neurons, but is released following detergent extraction that is required to expose the cytoskeleton for PREM analysis. In this scenario, all components remain intact but lose their alignment in the absence of tension. Thus, further investigation is needed to resolve discrepancy between PREM and STORM data.

Whether periodic actin rings in neurons have functional significance or simply result from longitudinal stretching of the submembrane network remains undetermined. On the other hand, separate functions have been defined for stable and dynamic populations of actin filaments in the AIS. When both actin filaments and microtubules are depolymerized in membrane-extracted hippocampal neurons, the AIS submembrane cytoskeleton remains completely intact and short stable actin filaments remain incorporated in the cytoskeletal network, although dynamic actin filaments are removed. Thus, we speculate that the short stable actin filaments are likely crucial for maintaining the structural integrity of the AIS submembrane cytoskeleton, probably by linking spectrin molecules, roughly similar to junctional complexes in erythrocytes. On the other hand, the longer dynamic filaments may play an essential role in the structural dynamics or plasticity of the AIS. This idea is supported by the finding that, in live hippocampal neurons treated with latrunculin B, the submembrane cytoskeleton appears damaged (containing holes or gaps) at AIS branch junctions where, under normal conditions, the cytoskeletal network appears to undergo “stretching” in response to tension exerted by the axonal branch [19]. Probably, dynamic actin contributes to a repair mechanism in instances where the cytoskeletal network is damaged due to breakage in response to mechanical stress. The AIS is also known to undergo changes in both position and length as a possible way to fine-tune neuronal excitability [64–66]. Dynamic actin filaments may also function in this type of AIS remodeling.

Together, recent studies using electron or subdiffraction fluorescence microscopy indicate that the submembrane cytoskeletal components form a sophisticated network in the AIS that is conceptually similar to the polygonal/triangular network of erythrocytes [16–20]. In this submembrane AnkG/*β*IV-spectrin/actin cytoskeleton, *β*IV-spectrin (with or without an *α*-spectrin partner) appears to arrange longitudinally along the AIS length, where it links adjacent actin rings. Some geometrical differences in the organization of the network components in neurons and erythrocytes may arise from different distribution of intracellular forces and distinct shapes of these cells. AnkG binds to the spectrin-actin network via its spectrin-binding domain and links the plasma membane to this network by binding ion channels and CAMs via its membrane-binding domain. The C-terminal region of AnkG extends into the AIS cytosplasm where it may link the spectrin-actin network to intracellular cytoskeletal components.

### 5.3. Contribution of the AIS Submembrane Cytoskeleton to the Diffusion Barrier

Evidence of an AIS diffusion barrier first derived from the observation that fluorescent lipids in the plasma membrane of cultured hippocampal neurons are incapable of spreading from the distal axonal domain, where they diffuse freely, to the somatodendritic domain [6]. Subsequent studies revealed that diffusion of plasma membrane proteins across the AIS is also blocked [5, 67]. The restricted mobility of both proteins and lipids in the AIS is thought to rely on the submembrane cytoskeleton. Disrupting the actin cytoskeleton by latrunculin B treatment increases the intrinsic mobility within the AIS of L1 CAM (a transmembrane protein) and Thy-1 (a GPI-anchored protein, which therefore spans only the outer leaflet of the plasma membrane) [5], as well as of single fluorescently labeled lipids tracked by live cell imaging [62]. Furthermore, when beads attached to L1 or Thy-1 were dragged using optical tweezers in the AIS plasma membrane, they moved longer distances after exposure to latrunculin B. Based on these observations, Nakada et al. (2003) proposed the anchored-protein picket model, implicating the submembrane cytoskeleton as a key contributor to barrier function [62].

According to this model, the mobility of plasma membrane components, both proteins and lipids, is largely restricted by transmembrane proteins, or “pickets,” that are themselves immobilized through anchorage to the underlying actin cytoskeleton, or “fence” [6, 62]. In current terms, the role of the fence should be assigned to the submembrane meshwork dominated by spectrin and AnkG with a small amount of actin, rather than to a dense actin meshwork, as proposed by the authors. The anchored proteins, or pickets, likely correspond to ion channels and CAMs which cluster in the AIS plasma membrane and attach to the submembrane cytoskeleton through AnkG. In support of this idea, it has been shown that the barrier forms only after AnkG-dependent immobilization of Nav channels, and AnkG accumulation alone is insufficient for barrier function [67]. Components of the AIS submembrane cytoskeleton that associate with plasma membrane lipids may also contribute, directly or indirectly, to the pickets. For example, AnkG is anchored to the plasma membrane at its N-terminal region via a palmitoyl anchor [68]. In addition, *β*IV-spectrin is thought to associate with plasma membrane phosphoinositides via its PH domain [69], and mice lacking *β*IV-spectrin PH domains exhibit nodes of Ranvier with impaired molecular and structural polarity [30, 70, 71]. The AIS plasma membrane lipid composition may also affect membrane fluidity and thus diffusion barrier function, but this aspect of AIS organization is largely understudied.

It appears that the barrier functions primarily through protein crowding within the AIS and that the specific organization of plasma membrane and cytoskeletal components may be irrelevant for barrier function. For example, if AIS organization was important for barrier function, then one would expect the barrier to form during or after AIS arranges into a periodic pattern. In contrast, available data show that the barrier is already functional between 7 and 10 days* in vitro* (DIV) [62], whereas the AIS becomes periodic only at 12 DIV [18]. However, potentially different culture conditions in these unrelated studies make interpretation difficult, so it would be helpful to address this question within the same study. Regardless of an effect of periodicity, the submembrane cytoskeleton appears to contribute to the AIS diffusion barrier primarily by providing a platform on which proteins are anchored, thereby allowing them to restrict the mobility of plasma membrane components through either steric hindrance or direct interactions, potentially independent of AIS-specific organization [61, 62].

Would axons actually lose polarity if the AIS submembrane cytoskeleton is disrupted? In this respect, experiments disrupting actin filaments are not fully informative, because of critical impact on many other aspects of neuronal function that would confound the results. On the other hand, targeting an AIS-specific protein, for example, AnkG, would less likely introduce secondary consequences that might also affect polarity. Depletion of AnkG in cultured hippocampal neurons not only dismantles the AIS but also causes axons to partially lose their identity: they acquire both membrane and cytoplasmic proteins that are normally found exclusively in the somatodendritic domain [72]. More importantly, these axons also develop spines and excitatory postsynaptic densities within their proximal regions [72], a phenomenon also observed in neurons lacking AnkG* in vivo* [73]. These data support the idea that AnkG-dependent submembrane cytoskeleton in the AIS is important for neuron polarity. However, disruption of axon identity is only partial (i.e., proximal) following AIS disassembly, because more distal axon regions retain axonal markers. Therefore, additional investigation is required to determine whether a distal barrier exists in axons or whether other mechanisms contribute to maintenance of axon identity in this region. Since knockout of *β*IV-spectrin resulted in partial loss of Nav channels and AnkG from the AIS and nodes of Ranvier [30, 55, 56, 74], it is also expected to contribute to neuron polarity, but experiments directly testing a role for *β*IV-spectrin in maintaining axonal or somatodendritic identity are lacking.

## 6. Cytoskeleton of the Inner AIS Shaft

Like elsewhere in neurons, the cytoplasm of the AIS contains microtubules, neurofilaments, and actin filaments, but differences exist in the AIS in terms of the structure and organization of these filaments. In addition, since the unstructured C-terminal tail of AnkG extends into the AIS cytoplasm, where it performs potential interactions with cytoplasmic components, we treat it here as a constituent of the inner AIS shaft.

### 6.1. AnkG C-Terminal Tail

Cross-sectional views of the AIS obtained by 3D STORM imaging demonstrated that the C-terminal tail domain of AnkG extends below the submembrane spectrin-actin network to an average depth of 26 nm relative to the *β*IV-spectrin C-terminus and a maximum depth of ~140 nm, into the intracellular AIS shaft [16]. The depth of ~140 nm likely reflects the maximal intrinsic length of AnkG molecules in cells, as suggested by immunogold PREM data revealing some AnkG molecules as thin curvy filaments that can reach up to ~150 nm in length [19, 75]. By penetrating deep into the AIS cytoplasm, AnkG may interact with cytoplasmic binding partners, such as microtubule fascicles. However, instances of deeply penetrating AnkG tails were infrequent, suggesting that they rarely reach microtubule fascicles, which according to electron microscopy data are present throughout the AIS interior [9, 10, 76]. In contrast, based on 3D STORM data, microtubules were reported to display an average depth of 85 nm below the plasma membrane [16], suggesting predominantly peripheral localization of microtubules in the AIS, which seems to conflict with electron micrographs of thin tissue sections [9, 10, 76]. Future analyses are needed to resolve this discrepancy.

### 6.2. Microtubules

In both the AIS and distal axon, microtubules are uniformly oriented with their plus ends pointed toward the axonal tips, whereas in dendrites microtubules generally have mixed orientation, with their plus ends facing either toward or away from the soma [77–79]. Cross sections through the distal axon and dendrites reveal microtubules spaced 25 nm and 65 nm apart, respectively [80]. This difference in interfilament spacing is likely due to interaction with different microtubule associated proteins (MAPs), which are known to regulate microtubule bundling. MAP1B and Tau mainly decorate microtubules in axons [81–84], whereas MAP2 localizes specifically to the somatodendritic domain [85–87]. Expression of Tau and MAP2 in nonneuronal cells results in microtubules with an interfilament spacing of 25 nm and 65 nm, respectively, similar to that observed in axons and dendrites [80].

In the AIS, microtubules are more tightly packed into fascicles [9]. A microtubule fascicle can be defined as a collection of individual microtubules that align parallel with one another and are closely cross-linked into a bundle. Unlike the distal axon or somatodendritic domain, microtubules within AIS fascicles are each coated by a cloud of dense material, likely corresponding to MAPs or other regulatory proteins, and connected to neighboring microtubules by thin fibrils that create 12 nm bridges [9, 10]. These bridges link the parallel microtubules together into a series, such that (in cross section) each fascicle appears to form a branched or linear chain. Depending on the neuronal type and the diameter of the AIS, the number of fascicles can range from about 3 to 7, and the number of individual microtubules within each fascicle can vary from 2 to 25 [9]. In electron micrographs, AISs often appear to contain 1–3 individual microtubules that are not fasciculated [9]. Like microtubules in fascicules, these individual AIS microtubules are surrounded by a cloud of dense material and often appeared to contain one or two fibrils extending from the surfaces [10], thus indicating that individual AIS microtubules are likely structurally similar to microtubules within fascicles.

Fascicles may be considered the main distinctive feature of the AIS because they are absent from all other neuronal regions, even nodes of Ranvier, where microtubules are evenly dispersed and display a loosely bundled or nonbundled arrangement [9]. The lack of fascicles in nodes of Ranvier may be unexpected since nodes share many features with the AIS, including a similar membrane and submembrane protein composition [8, 88, 89] and periodic organization of the submembrane cytoskeleton [17]. On the other hand, nodes of Ranvier in contrast to the AIS have not been implicated in polarity function, suggesting that fasciculation of microtubules may be specifically relevant to the role of the AIS in determination of neuron polarity.

How is a microtubule fascicule generated in the AIS? A recent study identified the axonal MAP tripartite motif containing (TRIM) protein, TRIM46, as a potential key contributor to formation and maintenance of AIS microtubule fascicles [90]. In particular, when expressed in HeLa cells TRIM46 induces bundles comprising ~5–9 individual microtubules spaced similarly to microtubules in AIS fascicles [10]. In various neuronal types, TRIM46 localizes specifically to microtubules in the proximal axon, but not in nodes of Ranvier. In addition, during early neuronal development, TRIM46 accumulates in a single neurite (i.e., the future axon) even before it can be morphologically identified as an axon [90]. Such an early arrival of TRIM46 to the future axon is consistent with the PREM data showing that microtubule fasciculation is already apparent by 3 DIV [19]. However, it is still unclear whether AIS-specific fascicles are unbundled or fail to form in the absence of TRIM46, although TRIM46 knockdown inhibits early stages of neuron polarization and affects vesicular trafficking in axons [90]. Perhaps the primary function of TRIM46 is to organize AIS microtubules into uniform parallel arrays, an idea that is supported by data showing microtubules displaying mixed orientation in the proximal axon of TRIM46-depleted hippocampal neurons [90].

Additional promising candidates for generating or maintaining microtubule fascicles include the microtubule end binding (EB) proteins, members of a family of microtubule plus-end tracking proteins (+TIPs), that were found to accumulate in the AIS [91, 92]. Normally, EB proteins dynamically bind to the plus ends of growing microtubules where they regulate microtubule growth. Both generic EB1 and neuron-specific EB3 display plus-end tracking activity in axons and dendrites [78, 93–97]. In contrast, EB proteins in the AIS are thought to be stably bound along the entire length of microtubule fascicles [91]. However, reports on the timing of EB protein localization in the AIS during development are conflicting. Initial experiments showed EB association along the microtubule lattice only when the AIS is mature [92], indicating that EB proteins likely do not initiate fascicle formation, which occurs very early during AIS development [19], but may instead contribute to maintaining fascicles. In a more recent study, however, EB proteins were found to localize to proximal axons as early as 2 DIV where they interact specifically with the 480 kD AnkG isoform and are important for AnkG accumulation and stability at the early AIS [98]. Thus, cooperation between EB proteins and 480 kD AnkG may specify the position of the AIS during early development. Further investigation is required to resolve the developmental timing of EB protein accumulation at the AIS and to determine whether EB proteins have a direct role in microtubule fasciculation.

### 6.3. Actin

Aside from forming periodic rings as in the submembrane cytoskeleton, actin can form other stuctures within the AIS and axon. For example, in axons, actin is often found organized in small patches of branched-filament meshworks [99]. These small actin patches serve as precursors to filopodia, which can in turn give rise to axonal, and possibly AIS, branches [17, 19, 99, 100]. There is also evidence that AIS actin patches may additionally provide scaffolding support for presynaptic boutons, since the patches colocalize with presynaptic markers [17, 101, 102]. Some actin patches are also thought to function in vesicular transport (discussed below) [103]. Subdiffraction microscopy also showed that actin filaments appear to form bundles along the AIS and distal axon that vary in length and localization depending on the age of the neuron [17, 20]. However, to what extent actin patches and bundles reside in the AIS interior or whether they mostly represent surface structures remains to be shown.

### 6.4. Neurofilaments

Neurofilaments are a class of intermediate filaments and display a diameter of ~10 nm. Neurofilaments are abundant in axons where they play an important role in regulating axon diameter, which in turn affects the velocity of electrical signal conductance [35, 104–108]. This ability of neurofilaments to control axon diameter, and therefore signaling, depends on the density of and spacing between filaments. The interfilament spacing is determined by phosphorylation of neurofilament subunits, resulting in small extensions with the appearance of hair-like “side-arms” that bind neighboring filaments [109–111]. In the distal axon, neurofilaments are arranged longitudinally in parallel with one another, exhibit an interfilament spacing of 33–49 nm [112, 113], and often appear curvy due to their intrinsic flexibility. In contrast, neurofilaments in nonmyelinated axon regions, probably, including AIS, are more closely bundled [114] and form straight parallel arrays [9, 10]. More linear AIS neurofilaments may generate if they are under greater tension than in the distal axon. Alternatively, neurofilaments in the AIS may straighten and be more closely spaced due to their different properties because AIS neurofilaments are known to be less phosphorylated than in the distal axon [114]. Although very little attention has been given to the organization and structure of AIS neurofilaments, they may be critical for proper AIS function. For example, changes in the spacing and density of neurofilaments have been associated with both alterations in AIS diameter and early pathological changes in motor neuron disease [115].

Clearly, AIS intracellular cytoskeleton components exhibit organizational and structrural features that are distinct from distal axon and somatodendritic regions. Straight parallel neurofilaments and microtubule fascicles present the most distinct structural features of the AIS interior cytoskeleton. However, the mechanistic basis underlying formation of these stuctures is not fully clear.

### 6.5. Role of the AIS Cytoplasmic Cytoskeleton in the Intracellular Selective Filter

To be an effective regulator of neuron polarity, the AIS must control not only the diffusion of membrane components in the proximal axon, but also the cytoplasmic distribution of axonal and somatodendritic constituents. This function is attributed to the intracellular selective filter, a multifaceted but not fully understood mechanism that allows only certain organelles and molecules to enter the axonal domain, while preventing the entry of other components destined for dendrites [116]. For example, live cell imaging of fluorescently tagged proteins shows that axonal constituents can enter both axonal and dendritic domains, while somatodendritic proteins are blocked from invading axons [117, 118]. The intracellular selective filter can be divided into two distinct processes that involve (1) active directional transport and (2) a passive cytoplasmic diffusional barrier. Both of these processes are dependent on the integrity of microtubules and actin filaments and possibly other components in the AIS cytoskeleton.

#### 6.5.1. Active Directional Transport

Microtubules are famous for their function in vesicular transport. Accordingly, microtubules in the AIS are thought to contribute to the filter by forming oriented tracks for polarized vesicular transport by the molecular motors kinesin and dynein. Since dynein moves along microtubules toward their minus ends and when artificially linked to organelles can target them to dendrites [119], it has been hypothesized that this motor may be important for excluding dendritic proteins from axons [120]. In support of this view, dynein activator nuclear distribution element-like 1 (NDEL) accumulates at the AIS in an AnkG-dependent manner, along with the NDEL binding partner LIS1 [121]. NDEL-based dynein activation in the AIS functions to reverse traffic of somatodendritic vesicles, since deletion of NDEL or LIS1 results in the axonal entry of dendritic cargo, including transferrin receptor and the AMPA receptor subunits GluR1 and GluR2 [121]. Kinesin motors may help to selectively deliver axonal constituents, because they generally move toward the plus ends of microtubules, resulting in transport from the soma into axons. Indeed, kinesin KIF5B drives the axonal transport of the synaptic protein VAMP2 that functions in axons. However, some kinesins also transport dendritic cargo. For example, kinesin KIF17 transports the NMDA receptor subunit NR2B that functions in dendrites [122]. Nonetheless, the situation is not so simple, because KIF5 (e.g., KIF5B) has been shown to drive transport of both axonal and somatodendritic proteins [122]. Song et al. (2009) addressed the question of AIS filter selectivity for motor proteins and their associated cargo by analyzing the transport of KIF5B and KIF17 chimeras [116]. They revealed that permissive vesicular transport toward the axon depends more on features of the motor-cargo complex rather than the type of motor alone [116].

Additional determinants of protein trafficking across the AIS may involve features of the microtubule tracks themselves. For example, Nakata and Hirokawa, (2003) revealed preferential binding of AIS microtubules by KIF5 [91]. This preference was suggested to involve AIS-specific posttranslational modifications (e.g., acetylation, detyrosination, and polyglutamylation) or increased GTP presence in the AIS microtubules (reviewed in [120]). Some AIS-specific MAPs that constitute the dense coating of microtubules in the AIS may also contribute to track-dependent selectivity. Furthermore, formation of microtubule fascicles in the AIS may also serve as a selectivity signal. For example, a specific arrangement of motors on the axonal cargo may take advantage of fasciculated microtubules, whereas a different arrangement of motors on the dendritic cargo may be incompatible with fasciculated tracks. It remains to be shown whether fasciculated and individual AIS microtubules have distinct roles in protein trafficking across the AIS.

Actin filaments may contribute to the intracellular selective filter by serving as tracks for myosin motors. Myosin V that walks toward the barbed ends of actin filaments and myosin VI that travels toward the pointed ends play important roles for transporting somatodendritic and axonal proteins, respectively, to their proper locations in neurons [123, 124]. Moreover, Watanabe et al. observed that nonspecific cargo linked to myosin VI proceeds unimpeded from the cell body through the AIS toward the distal axon, whereas vesicles linked to myosin V are stalled in the AIS and prevented from entering the distal axon, or occasionally reverted back to the cell body [103]. These data suggest that actin filaments within the AIS may be uniformly oriented with their barbed ends facing the soma, which would allow them to act as a selective filter by excluding somatodendritic proteins from entering the axon through myosin V, while simultaneously selecting axonal proteins to enter the axon via myosin VI [118]. However, polarized actin tracks in the AIS have not been directly visualized. Correlation of vesicle movements with localization of actin filaments examined by fluorescence microscopy after fixing the cells revealed that vesicles linked to myosin V were stalled at discrete actin patches. By electron microscopy, analogous patches appeared as elaborated networks of nonaligned intertwined actin filaments [103]. This morphology is not fully consistent with the idea of longitudinally oriented tracks with uniform polarity, although it is conceivable that these patches may trap vesicles. Direct determination of actin filament polarity using decoration with myosin subfragment 1 showed that actin filaments in the AIS have random orientation and thus are not expected to support polarized transport [19]. Notably, both studies use PREM and thus primarily visualize surface structures, raising a possibility that the cytoplasmic actin filaments predicted to form oriented tracts remain undetected in the AIS interior. One argument against this possibility is that oriented actin tracks are still undetectable in AIS regions having relatively sparse submembrane cytoskeleton, where examination of the AIS interior is possible. Furthermore, D'Este et al. (2015) detected a population of somatodendritic actin filament bundles, which end at the proximal border of the AIS and suggested that this “organizational border” may also (or instead) support the selective filter by causing the abrupt arrest of dendritic cargo attempting to enter the axon [17], but this idea remains to be tested.

#### 6.5.2. Passive Cytoplasmic Diffusion Barrier

The actin cytoskeleton also contributes to the intracellular diffusion of specific cargo through the AIS. By tracking fluorescently labeled dextrans of various molecular weights in hippocampal neurons, Song et al. (2001) showed that large dextrans are prevented from entering the axon between 3 and 5 DIV, during the time of AIS formation [116], but can diffuse across the AIS after depolymerization of actin filaments by latrunculin A. These data suggested the presence of a size-selective sieve in the AIS interior that depends on integrity of the actin cytoskeleton. However, specific configuration of the sieve is unclear. If it is actin filaments that make a cytoplasmic size-exclusion sieve in the AIS cytoplasm, they should be sufficiently dense and thus significantly enriched in the AIS, which is not the case [19]. Interestingly, RNAi-mediated knockdown of AnkG also allows for dextran diffusion across the AIS. A proposed explanation for this effect is that the C-terminal cytoplasmic tail of AnkG extends into the AIS interior, where it links to microtubules to form a sieve that blocks the entry of macromolecules into the axon [120]. However, further investigation is needed on this point since the AnkG C-terminus penetrates on average only ~26 nm into the AIS interior [16], which seems insufficient for making a sieve with an appropriate density by interacting with microtubule fascicles located deep in the AIS cytoplasm. Therefore, it remains unclear how disruption of AnkG and actin filaments produces similar phenotypes in terms of regulating the selective filter.

In addition, microtubules may contribute to maintaining asymmetric accumulation of proteins in axons via a sequestration mechanism, rather than through motor-dependent traffic. Thus, Li et al. (2011) showed that when microtubules are depleted, the axonal MAP Tau, which is normally bound to microtubules in axons, passes through the AIS and invades the somatodendritic domain, without affecting AnkG clustering [125]. These data suggest that microtubules may retain some axonal proteins and prevent them from retrograde diffusion into the somatodendritic domain [125].

Thus, the existing observations show that the intracellular cytoplasmic filter in the AIS contributes to maintaining the polarized distribution of somatodendritic and axonal components in neurons. The underlying mechanisms apparently include motor-driven vesicular transport along both microtubule and actin tracks, as well as an internal diffusion barrier that plays a more passive role. However, much remains to be understood about specifics of these mechanisms. Indeed, with the exception of the AIS-bound NDEL-based activation of dynein, much of what is known regarding active directional transport in the AIS has been extrapolated from roles of microtubules and microtubule-based motors in the distal axon. Further investigation is required to firmly establish the existence of a bona fide cytoplasmic selective filter in the AIS that is distinct from other neuronal domains.

## 7. AIS Cytoskeletal Assembly and Maintenance

### 7.1. Assembly

The AIS cytoskeleton is built during early neuronal development following axon specification. Based on observations* in vitro*, neurons develop through several stages that can be identified by distinct morphological events [126]. When neurons are plated on a substrate, they start out as symmetrical cells containing a round soma with abundant lamellipodial protrusive activity at the perimeter (stage 1). Eventually, these protrusions give way to multiple short processes of about equal length called neurites (stage 2). After approximately 1.5 days* in vitro* (DIV), the neurons become polarized as one of the neurites outgrows the others and differentiates (molecularly and functionally) to become an axon (stage 3). By 4 DIV, the remaining neurites begin to slowly elongate and develop into highly branched dendrites (stage 4). After 7 DIV, the axon and dendrites progressively mature to form synapses and the neural circuitry (stage 5).

After axon formation (early stage 3), AnkG begins to cluster in the proximal axonal region, indicative of early AIS assembly [127]. Structural analysis by PREM of AIS development revealed that microtubule bundling appears as the first sign of AIS assembly in early DIV hippocampal neurons before signs of submembrane cytoskeletal assembly become detectable [19]. The causative relationship between AnkG accumulation and microtubule bundling remains unclear. Early formation of fascicles by TRIM46 or EB proteins may initially help stabilize AnkG in the AIS and promote its clustering [128, 129], if the AnkG C-terminal tail preferentially interacts, directly or indirectly, with bundled microtubules. In support of this idea, the C-terminal tail specific for the large, 480 kD AnkG isoform is necessary for the accumulation and stability of AnkG in the AIS of early hippocampal neurons and subsequent assembly of the AIS [98]. Alternatively, AnkG may contribute to fasciculation of microtubules, because AnkG is capable of both oligomerizing and binding microtubules, either directly [11] or indirectly (e.g., through EB proteins [98]), and fascicles fail to form in AnkG-depleted cerebellum axons [73]. Together, these observations suggest a possible cooperative relationship between early AnkG clustering and microtubule fasciculation.

**Figure 3:**
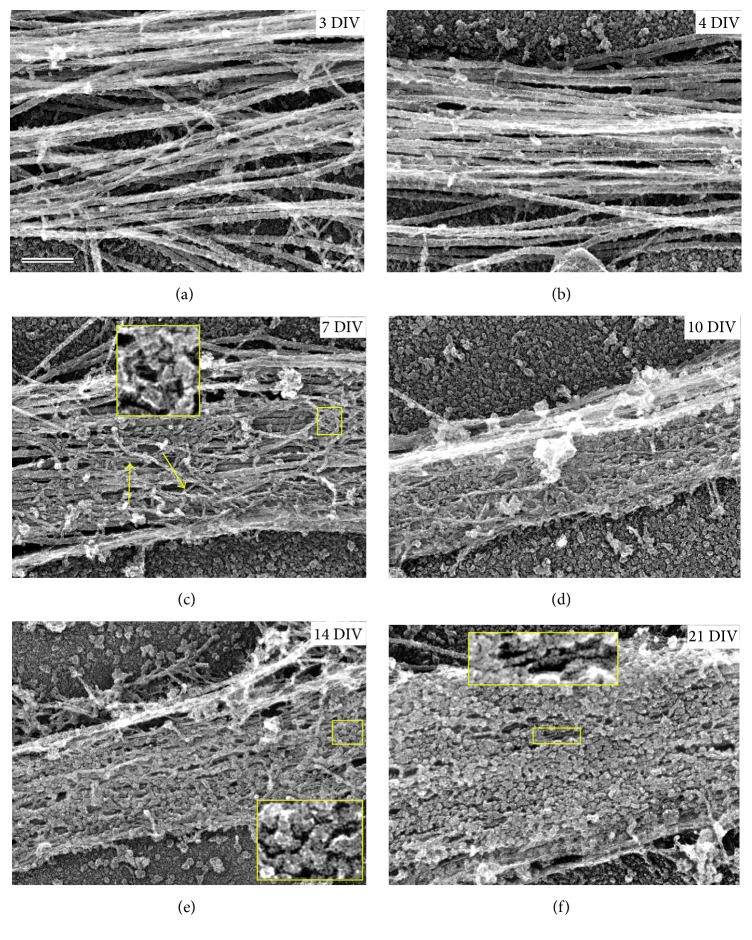
PREM images showing different stages of assembly of the AIS coat in hippocampal neurons. ((a)-(b)) Proximal axons of early 3 and 4 DIV neurons showing microtubules that are either loosely aligned (a) or tightly bundled (b), respectively. (c) Proximal axon of 7 DIV neuron displaying a loose fibrillar (arrows) coat containing sparse globules (inset) covering the microtubules. (d) Proximal axon of a 10 DIV neuron containing a dense fibrillar-globular coat. (e) A 14 DIV neuron displaying a dense coat containing mostly globules (inset) and few visible fibrils. (f) A 21 DIV neuron revealing a mature coat dominated by globules, although few parallel fibrils are visible (inset).

PREM observations at different time points during neuron development revealed that the AIS submembrane cytoskeleton undergoes structural reorganization over time (Figure 3), which likely reflects arrival of additional AIS proteins, as well as their spatial rearrangement. Immunogold staining of individual AIS proteins made it possible to link individual shapes within the AIS submembrane cytoskeleton to specific proteins [19]. When the submembrane cytoskeleton in the AIS begins to assemble over the bundled microtubules at 3 DIV, it initially adopts a sparse meshwork consisting of thin fibrillar elements and actin filaments. The thin fibrils likely represent a mixture of AnkG, *β*IV-spectrin, and some CAM molecules, all of which have wavy linear shapes. Subsequently, globular structures that display a range of shapes and sizes gradually accumulate in the AIS cytoskeleton. Many of these globules, especially those with donut-like shapes, likely represent various ion channels. They begin to noticeably appear at 7 DIV and become prevalent surface components of the AIS by 21 DIV. Interestingly, some globules also appear in elongated clusters that likely correspond to AnkG molecules associated with AnkG binding partners, such as ion channels. At 21 DIV, the extreme density of putative ion channels on the AIS surface renders the fibrillar structures nearly invisible, except in small spaces between globules. However, these “windows” reveal the transition of thin fibrils from the mixed/meshwork-like configuration to a striking longitidinal orientation between 14 and 21 DIV. This rearrangement may reflect a transformation of relaxed wavy spectrin molecules to a stretched linear conformation, which likely accompanies the appearance of the periodic organization observed by light microscopy [18].

The transition from meshwork to periodic organization in the AIS submembrane cytoskeleton was investigated by Zhong et al. (2014), who showed that *β*II-spectrin, an isoform enriched in distal axons, has an important role in formation of AIS periodicity [18]. They found that *β*II-spectrin adopts a periodic arrangement at the site of future AIS as early as 2 DIV, before arrival of AIS components. The *β*II-spectrin periodicity then propagates in the distal direction. AnkG and *β*IV-spectrin that subsequently arrive to the AIS adopt a periodic organization only after 12 DIV, which occurs in parallel with loss of *β*II-spectrin fluorescence intensity and periodicity in the proximal axon [18]. Additionally, knockdown of *β*II-spectrin in young neurons before the AIS is established partially impairs *β*IV-spectrin accumulation and periodicity. These data suggest that *β*IV-spectrin and AnkG incorporate into a preformed periodic template made by *β*II-spectrin in the AIS submembrane cytoskeleton. Because the isoforms of spectrin are exchanged in this process and AnkB is dispensable for *β*II-spectrin organization in axons, it seems that memory of periodicity resides in the actin-containing component of the *β*II-spectrin-based periodic structure. Notably, adducin is also gradually lost from the AIS during its maturation, suggesting that actin filaments in the periodic network may also change their partners during AIS development. An interesting speculation is whether this template mechanism is more important for certain aspects of AIS submembrane cytoskeletal organization than AnkG, even though AnkG plays a key role in recruiting AIS components. One possible scenario is that AnkG operates primarily to recruit and retain AIS components, while *β*IV-spectrin (in conjunction with *β*II-spectrin) helps to fine-tune the submembrane cytoskeletal architecture into the periodic configuration.

It is generally agreed that AnkG is a master organizer of the AIS, but a major unresolved question is how AnkG itself gets recruited to the proximal axon to start AIS assembly. It was initially proposed that AnkG targeting involves multiple domains within the molecule that cooperate with each other [130]. Subsequently, He et al. (2012) showed that S-palmitoylation at cysteine 70 within the AnkG N-terminal membrane-binding domain is particularly required for targeting of AnkG to the AIS, as well as to lateral membranes in epithelial cells [68]. Cooperation with EB proteins appears as another potential mechanism to specify the site of AnkG clustering and AIS localization [98].

Galiano and colleagues proposed a different model, an exclusion mechanism, for AnkG accumulation in the proximal axon [127]. According to this study, AnkG initially has a broad distribution in the axon, whereas components of the generic axonal submembrane cytoskeleton (AnkB, *α*II-spectrin, and *β*II-spectrin) predominantly concentrate in the distal end of the axon. As development of the neuron progresses, this generic cytoskeleton expands toward the soma where its advancing boundary forces the AnkG-containing submembrane cytoskeleton to concentrate in the proximal axon [131]. Supporting this idea, depletion of AnkB, *α*II-spectrin, or *β*II-spectrin results in the redistribution of AnkG and *β*IV-spectrin to the distal axon [127]. This exclusion mechanism seems incompatible with the study by Zhong et al. (2014), which places *β*II-spectrin-based submembrane cytoskeleton upstream of and as a requirement for the assembly of the AnkG/*β*IV-spectrin system in the proximal axon, and shows that it begins to assemble in the proximal, not distal, axon [18]. Possibly, the discrepancy between the two studies exists due to differences of focus on periodic [18] versus total [127] pools of these cytoskeletal components. Additional investigation is required to fully reconcile the findings of these two studies [18, 127].

Changes in actin filament organization during AIS development have recently received attention. In the AIS and distal axon, actin filaments assume different organizational forms, including actin rings, individual filaments, patches, and bundles. As judged by subdiffraction fluorescence microscopy, periodic actin rings in developing neurons are formed only at 5 DIV, even though periodicity of *β*II-spectrin is detectable at 2 DIV; this discrepancy is explained by potential technical difficulties of preserving actin in young neurons [18, 20]. PREM analysis of actin filament distributions in the AIS submembrane cytoskeleton showed that the two populations of individual actin filaments, long dynamic and short stable, exist during early neuron development and persist in mature AISs [19]. Actin patches that may support development of presynaptic structures [17, 101, 102] and axon branches [17, 19, 99, 100] form as early as 5 DIV throughout the axon [17]. Linear actin structures (interpreted as actin filament bundles) appear throughout axon development, although their abundance, length, and localization in the AIS vary depending on neuronal age, without adopting any easily recognizable pattern [17]. Thus, these observations suggest that the actin cytoskeleton undergoes various changes during AIS development. Although such changes may have functional consequences for different aspects of AIS structure and function, these questions remain largely unanswered.

### 7.2. Maintenance

Compared with the distal axon, the AIS cytoskeleton is considered an extremely stable structure in mature neurons. AnkG plays a major role in not only recruiting AIS components, but also keeping them localized to the proximal axon through direct interactions. The ability of *β*IV-spectrin to maintain proteins in the AIS is unclear because the evidence is conflicting. Initial studies reported that *β*IV-spectrin, particularly the full-length *β*IV∑1 isoform, is essential for AnkG and Nav localization at the AIS because these proteins fail to properly cluster at the AIS in neurons lacking this spectrin isoform [30, 55]. However, a later study showed that both AnkG and Nav remain localized to the AIS after *β*IV-spectrin depletion [35]. Interestingly, membrane proteins, such as CAMs, can also contribute to maintaining AIS components. For example, although the AIS fully assembles in the absence of neurofascin* in vitro* and* in vivo*, the long-term clustering of its components requires neurofascin [72, 132]. Finally, non-AIS factors may also contribute to maintaining AIS protein localization since, for example, the distal AnkB/*α*II-spectrin/*β*II-spectrin network can act as a barrier that restricts AIS components to the proximal axon [127].

Proteins also appear to be stably maintained within the AIS network exhibiting very slow turnover. Both AnkG and *β*IV-spectrin (also ion channels and CAMs) are long-lived in the AIS, with half-lives of at least two weeks [72]. Furthermore, the periodic network in the AIS is well maintained, compared with distal axons, following actin and microtubule perturbation in live neurons [18, 20]. It is not fully clear whether this stability is due to intrinsic properties of the periodic network or some other factors, such as interactions of the network with microtubule fascicles. However, this latter possibility may be unlikely since the radial orientation of the C-terminal tail of AnkG remains unperturbed following disruption of microtubules [16]. Observations by PREM also indicate formation of a robust network among AIS components because its structure remains largely intact for several hours following extraction of the plasma membrane and concomitant removal of actin filaments and microtubules prior to chemical fixation [19].

## 8. Concluding Remarks

The AIS cytoskeleton has a unique structure in the proximal axon. Here we focused on the cytoskeletal architecture of two distinct regions of the AIS, beneath the plasma membrane and within the cytoplasm, and discussed how they contribute to the role of the AIS in maintaining neuron polarity. Investigation of the AIS at different developmental time points by electron and subdiffraction fluorescence microscopy has increased our understanding of how the AIS assembles into a sophisticated network (submembrane) or acquires distinct structural features (cytoplasmic). However, a full understanding of how specific proteins contribute to AIS cytoskeletal organization is still lacking. Therefore, it is essential to elucidate the arrangement and interactions of specific AnkG and *β*IV-spectrin isoforms and to determine their roles in the development, stability, and overall architecture of AIS cytoskeleton. In addition, it will be valuable to determine the significance of microtubule fascicles and to identify proteins involved in fascicle formation and maintenance. Since the AIS is fundamental to the ability of neurons to fire action potentials, it is important to determine whether changes in AIS architecture (e.g., during AIS development) relate to changes in neuronal excitability. It remains to be shown whether changes in AIS architecture are accompanied by alternations in AIS position or length, for example, in response to injury or disease.
